# Does rehabilitation setting influence risk of institutionalization? A register-based study of hip fracture patients in Oslo, Norway

**DOI:** 10.1186/s12913-021-06703-x

**Published:** 2021-07-09

**Authors:** Rina Moe Fosse, Eliva Atieno Ambugo, Tron Anders Moger, Terje P. Hagen, Trond Tjerbo

**Affiliations:** 1 Department of Health Management and Health Economics, University of Oslo, Blindern, PO box 1089, 0317 Oslo, Norway; 2 Department of Health, Social and Welfare Studies, University of South-Eastern Norway, Horten, Norway

**Keywords:** Hip fracture, Rehabilitation, Institutionalization, Nursing home placement, Healthcare use

## Abstract

**Background:**

Reducing the economic impact of hip fractures (HF) is a global issue. Some efforts aimed at curtailing costs associated with HF include rehabilitating patients within primary care. Little, however, is known about how different rehabilitation settings within primary care influence patients’ subsequent risk of institutionalization for long-term care (LTC). This study examines the association between rehabilitation setting (outside an institution versus short-term rehabilitation stay in an institution, both during 30 days post-discharge for HF) and risk of institutionalization in a nursing home (at 6–12 months from the index admission).

**Methods:**

Data were for 612 HF incidents across 611 patients aged 50 years and older, who were hospitalized between 2008 and 2013 in Oslo, Norway, and who lived at home prior to the incidence. We used logistic regression to examine the effect of rehabilitation setting on risk of institutionalization, and adjusted for patients’ age, gender, health characteristics, functional level, use of healthcare services, and socioeconomic characteristics. The models also included fixed-effects for Oslo’s boroughs to control for supply-side and unobserved effects.

**Results:**

The sample of HF patients had a mean age of 82.4 years, and 78.9 % were women. Within 30 days after hospital discharge, 49.0 % of patients received rehabilitation outside an institution, while the remaining 51.0 % received a short-term rehabilitation stay in an institution. Receiving rehabilitation outside an institution was associated with a 58 % lower odds (OR = 0.42, 95 % CI = 0.23–0.76) of living in a nursing home at 6–12 months after the index admission. The patients who were admitted to a nursing home for LTC were older, more dependent on help with their memory, and had a substantially greater increase in the use of municipal healthcare services after the HF.

**Conclusions:**

The setting in which HF patients receive rehabilitation is associated with their likelihood of institutionalization. In the current study, patients who received rehabilitation outside of an institution were less likely to be admitted to a nursing home for LTC, compared to those who received a short-term rehabilitation stay in an institution. These results suggest that providing rehabilitation at home may be favorable in terms of reducing risk of institutionalization for HF patients.

**Supplementary Information:**

The online version contains supplementary material available at 10.1186/s12913-021-06703-x.

## Background

Hip fracture (HF) is common among the elderly, and it is associated with increased morbidity and mortality [1], as well as substantial functional decline and long-term institutionalization [2]. Recovery after HF can take up to four months for upper extremity functioning in activities of daily living (ADLs), up to nine months for balance and gait, and up to one year for instrumental and lower extremity ADL functioning [3]. Furthermore, a large proportion of HF patients do not fully recover [4], especially individuals with poor premorbid health [2]. Other factors that undermine recovery include comorbidities, cognitive impairment, poor nutritional status, depression, and poor social support (for a review, see [2]). Many patients therefore require assistance with ADLs if they are to continue living comfortably and safely at home. Some important purposes of the rehabilitation of HF patients are therefore to identify individual goals in order to restore mobility and independence, and to facilitate return to the pre-fracture residence and long-term well-being [5]. Many seniors, including those in need of long-term care (LTC), wish to remain in their own home for as long as possible [6]. Being capable of living in one’s own home is often associated with higher levels of perceived independence and autonomy, and increased well-being [7]. Institutionalization, on the other hand, can have a negative influence on the quality of life of the elderly [8]. Institutionalization is considered an important risk factor for depression [9], and for older adults in LTC, loneliness and anxiety are also common problems [10].

In addition to resulting in a personal burden on the patient, HFs are also associated with considerable social and economic costs [11]. Previously, the focus was primarily on the short-term costs, and measures were taken that were aimed at reducing or containing the costs associated with the acute care for HF [1]. The index hospital admission is found to be the leading expenditure during the first year after HF [4], and length of stay (LOS) is the most important determinant [1]. Therefore, there has been a financial incentive to reduce the LOS and transition patients into lower levels of care—which has been the general trend. However, the economic consequences of a HF extend far beyond the initial hospitalization [1, 4, 12, 13]. For many patients, a large part of the costs is attributable to the rehabilitation phase [1] and centers around use of services in primary or community care settings. Furthermore, transferring patients from hospital to lower levels of care does not necessarily reduce the total costs of HF faced by society. On the contrary, it may represent a cost-shift from the hospital to other healthcare service providers [1, 14].

Given that long-term disability and increased need for healthcare services post-HF have major ramifications for patients’ well-being and the total cost of care [15], the need for cost-effective care that also safeguards patient outcomes is an important priority for healthcare systems under pressure. In particular, it is desirable for both the individual and the healthcare system to develop rehabilitative care that maximizes independence and allows the patient to live at home for as long as possible [16]. Although current HF management guidelines [5] emphasize the importance of multidisciplinary rehabilitation to help patients recover faster and regain mobility, the existing literature indicates that more research is still needed to identify the optimal setting and content of rehabilitation within primary care systems [17]. Providing healthcare services in patients’ homes—especially rehabilitation and similar interventions aimed at maintaining or improving functioning and independence—is believed to provide better opportunities for tailoring care and training to the patients’ everyday needs [16, 18, 19]. This in turn can potentially reduce the future risk of falls and other adverse events—such as institutionalization for long-term care (LTC) [18, 20].

In recent years, there has been an increasing interest in ‘reablement’ (also called ‘restorative care’) in several Western countries. In Norway, reablement care is known as ‘everyday rehabilitation’[21, 22]. Reablement is a time-limited and intensive intervention, that takes place in the patient’s home, and it is usually seen as an alternative to usual homecare. The service is aimed at individuals experiencing a functional decline, and the purpose is to help them relearn skills and regain confidence in everyday activities, so that they can—even after illness or injury—live independent and meaningful lives [23]. This is achieved by training and supporting the patients to perform tasks themselves, rather than receiving compensating help or having someone else perform the tasks for them, as is the case in conventional home nursing [19]. The evidence on the effectiveness of reablement is currently ambiguous, but promising and increasing—suggesting a favorable effect on patients’ physical functioning [24, 25], better health-related quality of life and reduced need for healthcare services [26], and increased probability of remaining at home [18]. Considering the potential advantages of home-based rehabilitation, this type of rehabilitation is presumably underutilized, given that the proportion of patients discharged home is highly variable [27], and it appears that patients are seldom rehabilitated at home [28]. In Norway, for example, many municipalities are still testing and developing the service on a small scale–despite the fact that the country’s health authorities place great emphasis on the future importance of home-based rehabilitation. Further investigation is also needed to determine whether rehabilitation at home can be a viable alternative to institution-based rehabilitation (e.g., in a nursing home). If so, further cost-savings could potentially be achieved, and institutional places and resources in primary care could be reserved for the group of patients in need of such support—a group that is expected to become larger as a result of the aging population [29]. Comparing the effects of different rehabilitation settings on patient outcomes and resource use is however problematic because different individuals have different risk profiles. Nevertheless, some evidence exist, suggesting that home-based and institution-based rehabilitation are comparable for HF patients in terms of both patient outcomes and cost-effectiveness [30–32].

Everyday rehabilitation (reablement care) is a model that has quickly become popular in Norway and the Nordic countries [21, 22, 33]. Some factors that have contributed to the increased attention to everyday rehabilitation and other home and community based services include: rising healthcare costs associated in part with the growing elderly population [34–36] and the need to curtail such costs as manifest in trends towards deinstitutionalization (e.g., shorter LOS [37–39], reductions in institutional places/capacity [35, 40]), older adults’ preference to live at home for as long as possible [35, 41], and an interest in promoting healthy/active aging [42–44]. Because Norway is a small country (5.37 million inhabitants [45]) with a large public healthcare sector, there is a need to shape the health services in a sustainable way that takes into account the aging population. In Norway, the responsibility for providing healthcare services to the population is divided between the primary and specialist health services. The state, by its four health regions, is responsible for the specialist health services – including hospitals. In order to receive treatment in the specialist health service, a referral from the general practitioner (GP) is required. The primary health service, on the other hand, consists of the healthcare services that are organized by—and located in—the municipalities. As of January 1, 2020, Norway has 356 municipalities. The health services offered in the local communities include GPs and other nursing and care services (e.g., physio- and occupational therapists; various institutions such as nursing homes, day centers and assisted living homes; and homecare services). It is within this health service organizational context that everyday rehabilitation services are being delivered to care recipients, predominantly older adults, living at home.

The aim of the current study was to examine whether receiving rehabilitation outside an institution (versus a short-term rehabilitation stay in an institution) was associated with a lower risk of being admitted to a nursing home for LTC. Institutionalization is an important outcome after HF, because it is a major driver of costs. We analyzed this using data from a subgroup of individuals in Oslo, Norway, who suffered a HF between 2008 and 2013. The sample consisted of individuals aged 50 years and older, who lived at home prior to the HF incident. The patients received rehabilitation either outside of or in an institution within 30 days post-discharge from hospital; and they lived either at home or in a nursing home at 6–12 months after the HF. Utilizing registry data allowed us to compare the risk of institutionalization between the two rehabilitation settings after having adjusted for patients’ individuals risk profiles, and for variation across Oslo’s boroughs. Importantly, we also adjusted for differences between patients in use of healthcare resources during the first five months post-discharge from hospital. This is crucial, considering that for community-dwelling individuals, the setting in which rehabilitation takes place can also affect later use of services—for example if home-based rehabilitation especially promotes competence in independent living.

## Methods

### Data

The analyses in this study are based on merged, individual level data for years 2008–2014 from five Norwegian registries. Specifically, data on use of primary healthcare services (see Table 1) and functional status as measured by ADLs comes from *Gerica*, which is Oslo municipality’s electronic patient record database. Data on age, gender, comorbidities, and hospital admissions (admissions related to HF was identified by International Classification of Diseases 10th revision (ICD-10) codes S72.*) comes from *the Norwegian Patient Registry (NPR)*. Data on education, income, and wealth are from Statistics Norway’s database, *FD-Trygd*; and mortality data are from *the Cause of Death Registry*. Statistics Norway linked the data from the different registries by use of the national personal identification number (PID). The PID was removed before the research data was handed over to the researchers.

**Table 1:** Unit prices (in NOK) and weights of municipal primary healthcare services

Service	Price ^a^ (per unit)	Price ^a^ (per hour)	Price ^a^ (per day)	Source	Weight
**Home services**: ^**b**^					
Practical assistance with daily activities		591,23		[48]	0,1860
Practical assistance, daily activities – training		640,35		[48]	0,2014
User directed personal assistant		417,00		[48]	0,1312
Day center/day offer		292,70		[48]	0,0921
Food delivery	97,91			[49]	0,0308
Safety alarm	24,48			[49]	0,0077
Respite outside institution/house		279,91		[48]	0,0880
Support worker		219,35		[48]	0,0690
Care benefit		245,63		[48]	0,0773
Home nursing		694,85		[48]	0,2186
Rehabilitation outside of institution/housing		842,18		[50]	0,2649
**Housing**: ^**c**^					
Sheltered housing			2269,48	[48] ^d^	0,7138
Other housing ^e^			2269,48	[48] ^d^	0,7138
**Institutional services**: ^**f**^					
Respite (in institution/housing)			3179,34	[48]	1,0000
Day stay in institution			1101,44	[49]	0,3464
Limited stay, treatment			2937,17	[49]	0,9238
Limited stay, rehabilitation			3071,99	[48]	0,9662
Limited stay, other			2937,17	[49]	0,9238
Long term stay in institution			2354,02	[48]	0,7404

*Notes*: ^a^ in Norwegian kroner, 2019 value; ^b^ Services are delivered in patients’ homes; ^c^ The user has signed a rental agreement and pays rent; ^d^ 90 % of the price of a long-term stay in an institution; ^e^ The municipality has the housing for health and care purposes; ^f^ The patient stays in an institution

In this study, HF incidents are the units of analysis. To be included in the study, a patient must not have been hospitalized with HF as the primary diagnosis during the 365 days preceding the index admission, which is the initial hospitalization for HF and is part of the index hospital episode [46]. The *index hospital episode* is the first inpatient treatment for HF during the calendar year, and it includes between-hospital transfers in which no more than one day has passed from the point of discharge to admission into the next hospital.

### Sample

**Fig. 1:**
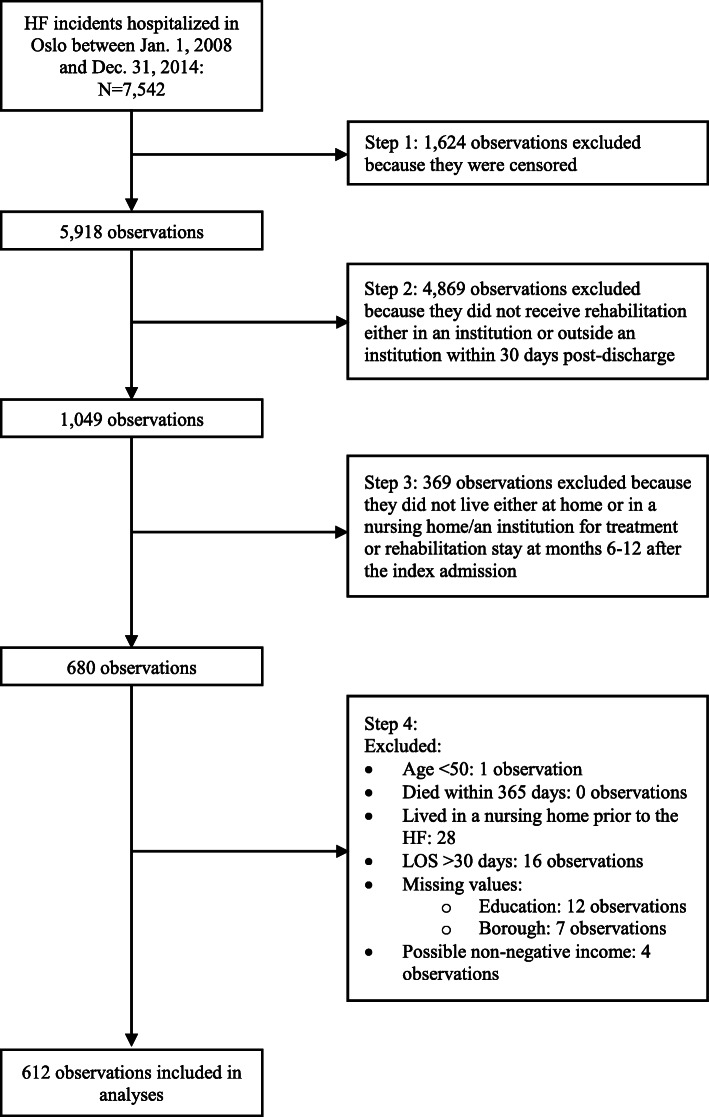
Flowchart of the exclusion of observations

The pre-analytic sample comprised 7,542 HF incidents in which patients had been hospitalized in Oslo between January 1st 2008 and December 31st 2014. We made some exclusions to arrive at our analytic sample of 612 incidents. This is illustrated in Fig. 1 and described in the following.

First, to define our sample and conduct our analyses, we needed patient data on the 90-day period before the index admission date, and on the 365-day period after the hospital discharge date. To ensure that all patients had data for these periods, the cases in which the patient was admitted to hospital prior to April 1st 2008 were excluded, as were cases where patients had a discharge date later than December 31st 2013. This resulted in the deletion of 1,624 censored observations.

Next, we excluded 4,869 cases in which the patient had not—within 30 days post-discharge—received rehabilitation either outside an institution or as a short-term stay in an institution.

Then, we identified the patients’ place of residence at 6–12 months after the index admission date. Cases in which the patient lived either at home or in an institution (i.e., in a nursing home or in an institution for treatment or rehabilitation stay) for the entire follow-up period were kept, and those in which the patient had varying residency (i.e., he or she lived both at home and in an institution during the period of interest) were excluded to reduce heterogeneity in our sample. These were 369 cases.

Finally, we excluded the following: (1) cases in which patients were under age 50, *n* = 1; (2) cases in which the patient died within 365 days from the index admission date, *n* = 0; (3) cases in which the patient resided in a nursing home throughout the 90-day period prior to the index admission, *n* = 28; (4) cases with a hospital episode LOS of more than 30 days, to reduce heterogeneity in the sample, *n* = 16; (5) cases with missing values on study measures (i.e., education and borough), *n* = 19; and (6) cases where we could not ascertain that the patient’s income was non-negative, *n* = 4.

The resulting analytic sample then consisted of 612 HF incidents across 611 unique patients who were 50 years or older. All patients were living at home before the HF, and they received rehabilitation in one of the two settings within 30 days after they were discharged from hospital. In the period from 6 to 12 months after the HF, they either lived at home or had been institutionalized for LTC.

### Measures

**Fig. 2:**
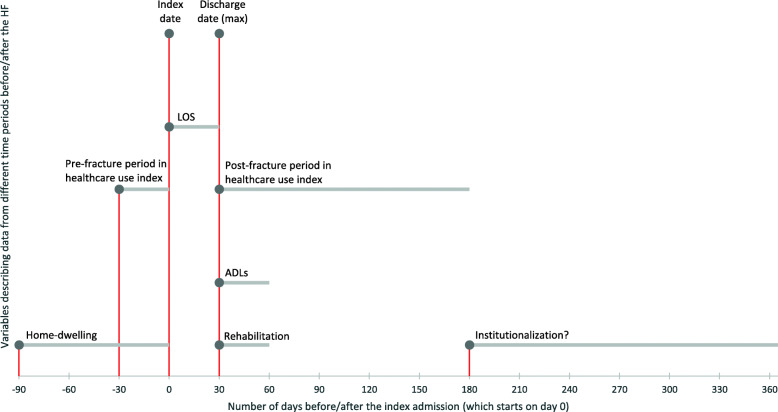
Timeline. A graphical representation of what periods and points in time our data measures

Figure 2 shows an overview of the events and time periods that the variables included in our analyses describe.

The dependent variable is *institutionalization*. It describes where the patient lives at 6–12 months after the index admission (1 = in nursing home or in an institution for a treatment/rehabilitation stay; 0 = home). This time period allows for patients to have completed their rehabilitation (rehabilitation outside of an institution is expected to last for 4–12 weeks [21], and a short-term rehabilitation stay in an institution is expected to last up to 12 weeks [47]), while also capturing the long-term effect on institutionalization.

The independent variable *rehabilitation* indicates in which setting the HF patient received rehabilitation services within 30 days post-discharge (1 = outside an institution; 0 = short-term rehabilitation stay in an institution). Rehabilitation outside of an institution means that the patient either received help from an intervention team that assessed his or her need for municipal care services and possibly provided rehabilitative training, or that the patient received everyday rehabilitation at home.

Risk adjustment was performed by controlling for the *gender* (1 = male, 0 = female) and *age* (in years at HF diagnosis, range: 50–101) of the patients, as well as their health characteristics, functional level, use of healthcare services, and socioeconomic characteristics, as described in the sections that follow.

Patients’ health characteristics are HF diagnosis and comorbidities. *HF diagnoses* were registered at the hospital based on ICD-10 codes (S72.0-S72.4 and S72.7-S72.9). The measure includes four categories: 1 = fracture of neck of femur, the reference group; 2 = pertrochanteric fracture; 3 = subtrochanteric fracture; 4 = others (fractures of: shaft of femur, lower end of femur, multiple fractures of femur, and other/unspecified parts of femur).

The presence of chronic conditions is captured in the binary variable *comorbidities* (1 = one or more comorbidities; 0 = no comorbidities). The variable comprises the following 16 disorders: acute myocardial infarction, hypertension, coronary artery disease, atrial fibrillation, cardiac insufficiency, diabetes mellitus, atherosclerosis, cancer, COPD and asthma, dementia, depression, Parkinson’s disease, mental disorders, renal insufficiency, stroke, and alcoholism. We dichotomized the variable because the data that were available to us included information on whether or not the patient had been hospitalized with any of the aforementioned illnesses as the primary or secondary diagnosis: a) during (at least^1^) the 90 days preceding the index admission for HF—for patients admitted in 2008 (N = 53); and (b) during the 365 days preceding the index admission for HF—for patients admitted after 2008 (N = 559). The prevalence of chronic conditions in our sample is therefore quite low because this measure only captures illness diagnosed and/or registered by the hospital, and not those diagnosed in other settings (e.g., GP offices)—data which we unfortunately lack access to.

The patients’ functional level in different areas is described by four categorical measures based on their ADL functioning: (1) *dependency in personal ADLs* (P-ADLs); (2) *dependency in instrumental ADLs* (I-ADLs); (3) *need for help with memory*; and (4) *need for help with social participation*. These variables are based on ADL assessments that took place after hospital discharge for HF. The scores we used to calculate ADL functioning are the first scores registered within 30 days after the discharge date. All four variables have three categories (described further below): 1 = low dependency (score ≤ 2), the reference group; 2 = high dependency (score > 2 and ≤ 5); and 3 = not assessed. The municipality obtained the ADL data, after (if) the patient applied for a health service that requires an assessment of the patient’s functional level. This requirement applies, for example, to health services delivered in the patient’s home, personal assistance, and institutional stays. If an assessment of ADL function has been carried out, an employee in the municipal health service has evaluated the patient’s ability to perform different activities, based on an overall assessment of the patient’s functional level at the time. The patient’s functional level for each activity was rated on a 6-point scale: 1 = poses no problem, no need for personal assistance; 2 = manageable, no need for personal assistance; 3 = moderate need for personal assistance; 4 = high need for personal assistance; 5 = full need for personal assistance; and 6 = not relevant. For our measure describing *dependency in P-ADLs*, we calculated the average score for the following six activities: taking care of your health, mobility indoors, personal hygiene, dressing and undressing, eating, and toileting. These activities are basic self-care tasks, which are essential for an independent life. For our measure describing *dependency in I-ADLs*, we calculated the average score for the following six activities: obtaining goods and services (e.g., shopping), making decisions in daily life, general housework, preparing food, mobility outdoors, and communication/communicating. These are more complex activities—allowing for active participation in the community. When we calculated these two average scores, we ignored scores that were lacking—meaning that the average score was calculated over the scores that were available to us. Patients for whom ADLs were not assessed lacked scores on all six P-ADLs/I-ADLs. We retained them in the “not assessed” category of these variables to make the comparisons between the other categories (low versus high ADL dependency) unambiguous, and to retain these HF patients in the analytic sample. The variable “*need for help with memory*” is based on the evaluation of the patient’s need for help with “memory/remembering/things”, and can be interpreted as a rough indicator of cognitive impairment. The variable “*need for help with social participation*” is based on the evaluation of the patients’ need for help to “develop/maintain/participate in a social network”, and can be interpreted as a rough indicator of social engagement, participation or isolation.

Two variables described patients’ use of healthcare services. We controlled for the *LOS* of the index hospital episode for HF (range: 2–30 days). To capture the scope of use of the municipal primary care services presented in Table 1, we created a *healthcare use index*. To create the index, we first weighted each service in Table 1 according to its relative cost, also presented in the table. Next, we summed up the weights of all the services the patient received during the first five months (150 days) post-discharge for HF, and deducted from it the sum of all the services received during the month (30 days) before the index admission. The derived healthcare use index (range: -1.3833 to 3.8191) therefore describes the change in the scope of service use between the pre- and post-fracture periods, where larger values on the index indicate a larger increase in the use of services.

Socioeconomic controls included *education* at the time of diagnosis (1 = primary education, the reference group; 2 = secondary education; 3 = tertiary education); gross *income* in Norwegian kroner (1 = 100,000-199,999; 2 = 200,000-299,999, the reference group; 3 = 300,000-399,999; 4 = 400,000+); and *wealth* in Norwegian kroner (1 = 0 or less, where negative values indicate debt; 2 = 1-199,999; 3 = 200,000-499,999; 4 = 500,000-999,999; 5 = 1,000,000+, the reference group). Data on income and wealth are from the year prior to the HF diagnosis.

Finally, we included a fixed-effect for each of Oslo’s *boroughs* (1 = Gamle Oslo, 2 = Grünerløkka, 3 = Sagene, 4 = St. Hanshaugen, 5 = Frogner, 6 = Ullern, 7 = Vestre Aker, 8 = Nordre Aker, 9 = Bjerke, 10 = Grorud + Stovner^2^, 11 = Alna, 12=Østensjø, 13 = Nordstrand, 14 = Søndre Nordstrand). Our regression models therefore generate within-borough estimates of the association between rehabilitation setting and risk of institutionalization. The fixed-effects adjust for unmeasured variation across the boroughs in factors such as the supply of nursing home places and other healthcare services.

### Statistical analysis

First, we generated the sample characteristics shown in Table 2. Then, we analyzed the risk of institutionalization in four logistic regression models. In the first model, we analyzed the effect of rehabilitation setting alone. In the second model, we included controls for patients’ gender and age, health characteristics, and functional level—and in the third, two variables describing patients’ use of healthcare services were added. Finally, in the fourth and full model, we also controlled for patients’ socioeconomic status (i.e., education, income, and wealth). All models included fixed-effects for Oslo’s boroughs. The variables were selected based on our review of existing literature, and were included regardless of how they performed in the models. We also reported a selection of goodness-of-fit measures to show the explanatory power of the models. To conduct the analyses, we used Stata SE/15.1 (StataCorp LLC, College Station, TX).

**Table 2:** Sample characteristics of HF incidents for patients hospitalized in Oslo between 2008 and 2013

	Place of residence at 6–12 months after index admission			
	**Home** **(*n* = 440)**		**Nursing home** **(*n* = 172)**	
	Mean or percent	SD ^a^	Mean or percent	SD ^a^
Rehabilitation outside institution (vs. in institution) ^b^	55.2 %		33.1 %	
Male (vs. female)	21.6 %		19.8 %	
Age (years)	81.0	(9.0)	86.0	(7.9)
One or more comorbidities (vs. none)	12.3 %		14.5 %	
HF diagnosis:				
Fracture of neck of femur	52.5 %		47.1 %	
Pertrochanteric fracture	36.1 %		40.7 %	
Subtrochanteric fracture	4.8 %		5.8 %	
Other ^c^	6.6 %		6.4 %	
P-ADL dependency: ^d^				
Low dependency	21.4 %		9.9 %	
High dependency	40.5 %		45.9 %	
Not assessed	38.2 %		44.2 %	
I-ADL dependency: ^d^				
Low dependency	8.4 %		2.9 %	
High dependency	53.0 %		50.0 %	
Not assessed	38.6 %		47.1 %	
Help with social participation: ^d^				
Low dependency	30.0 %		15.1 %	
High dependency	5.5 %		9.3 %	
Not assessed	64.5 %		75.6 %	
Help with memory: ^d^				
Low dependency	29.1 %		11.6 %	
High dependency	6.8 %		19.2 %	
Not assessed	64.1 %		69.2 %	
LOS (days) ^e^	11.2	(5.2)	12.0	(5.7)
Healthcare use index score ^f^	1.2	(0.6)	1.6	(0.9)
Education: ^g^				
Primary education	35.0 %		32.6 %	
Secondary education	49.1 %		49.4 %	
Tertiary education	15.9 %		18.0 %	
Income (in NOK): ^h^				
100,000-199,999	27.3 %		26.2 %	
200,000-299,999	38.6 %		37.8 %	
300,000-399,999	20.5 %		23.3 %	
400,000+	13.6 %		12.8 %	
Wealth (in NOK): ^h^				
≤ 0	0.2 %		0.6 %	
1-199,999	15.7 %		16.9 %	
200,000-499,999	17.5 %		19.8 %	
500,000-999,999	31.4 %		23.3 %	
1,000,000+	35.2 %		39.5 %	

*Notes*: ^a^*SD *standard deviation; ^b^ within 30 days post-discharge; ^c^ Fracture of: shaft of femur, lower end of femur, multiple fractures of femur, other/unspecified parts of femur; ^d^ within 30 days after hospital discharge for HF; ^e^ of the index hospital episode; ^f^ measures change in the scope of municipal healthcare services received between the first five months post-discharge and the month prior to the index admission; ^g^ in the year of HF; ^h^ in the year prior to the HF

## Results

### Sample characteristics

Table 2 shows descriptive characteristics of the study sample, by place of residence at 6–12 months after the index admission.

The proportion of patients who had received rehabilitation outside of an institution within 30 days post-discharge from hospital was 55.2 % in the group of non-institutionalized patients, and 33.1 % in the institutionalized group.

Males made up less than one quarter of the sample (21.6 and 19.8 %), and the mean age at the time of HF was higher among those who were institutionalized (86.0 years) compared to the individuals who continued to live at home (81.0 years).

The majority of the patients in the sample did not have any chronic conditions, but this finding must be seen in the context of how the variable is defined. With regard to the level of function after the HF, the two groups appeared quite different. In the group who lived at home at 6–12 months after the HF, 21.4 % had low dependency in P-ADLs, compared to 9.9 % in the group of patients who lived in a nursing home. The proportion of patients with low dependency in I-ADLs was also higher among patients living at home compared to those institutionalized for LTC (8.4 % vs. 2.9 %). Not surprisingly, patients living in nursing homes were more dependent on help with social participation and with memory.

The distribution of the type of HF was largely similar in the two groups. Fracture of the neck of the femur was the most common type of HF, accounting for almost half of all fractures considered here. Pertrochanteric fractures were also common.

The average LOS of the index hospital episode for HF was 11.2 and 12.0 days, respectively.

The healthcare use index, which measures the change in the scope of municipal healthcare services received during the pre- and post-fracture periods, was 1.2 (SD = 0.6) among patients who continued to live at home at 6–12 months after the HF, and 1.6 (SD = 0.9) among patients who were admitted to a nursing home for LTC. This indicates that, on average, all patients used more services after the HF and is consistent with expectations.

There were no major differences between the two groups with regard to educational level, income, or wealth. Most of the patients attained a secondary education, and about one in six earned a tertiary education. Still, a sizeable proportion of the patients only had a primary education, which is not surprising given this largely elderly sample who completed their education at a time when there was no universal access to education in Norway and attaining a high level of education was not the norm.

Supplementary file 1, Table A1 presents characteristics of the analytic sample and the excluded, censored observations (see step 1 in Fig. 1) on all study variables as well as for the exclusion criteria we used to define the analytic sample.

### Logistic regression

Table 3 shows the results from four logistic regression models. All models included fixed-effects for Oslo’s boroughs (regression coefficients are not shown).

**Table 3:** Logistic regression of institutionalization at 6–12 months after index admission for hip fracture on post-fracture rehabilitation setting ^a^

	Model 1		Model 2		Model 3		Model 4	
	**OR**^**b**^	**95 % CI**^**c**^	**OR**^**b**^	**95 % CI**^**c**^	**OR**^**b**^	**95 % CI**^**c**^	**OR**^**b**^	**95 % CI**^**c**^
Rehabilitation outside institution (vs. in institution)	0.26 ***	(0.16, 0.43)	0.31 ***	(0.18, 0.53)	0.43 **	(0.24, 0.77)	0.42 **	(0.23, 0.76)
Male (vs. female)			1.15	(0.69, 1.92)	1.12	(0.67, 1.89)	1.08	(0.62, 1.87)
Age (years)			1.08 ***	(1.05, 1.11)	1.07 ***	(1.04, 1.10)	1.08 ***	(1.04, 1.11)
One or more comorbidities (vs. none)			1.54	(0.85, 2.80)	1.61	(0.88, 2.93)	1.67	(0.92, 3.05)
HF diagnosis:								
Fracture of neck of femur			1.00		1.00		1.00	
Pertrochanteric fracture			1.16	(0.75, 1.78)	1.06	(0.68, 1.66)	1.08	(0.68, 1.70)
Subtrochanteric fracture			1.33	(0.52, 3.44)	1.25	(0.46, 3.40)	1.31	(0.47, 3.69)
Other ^d^			0.87	(0.37, 2.03)	0.78	(0.32, 1.91)	0.76	(0.30, 1.90)
P-ADL dependency: ^e^								
Low dependency:			1.00		1.00		1.00	
High dependency			1.12	(0.52, 2.40)	1.11	(0.51, 2.41)	1.08	(0.49, 2.37)
Not assessed			0.67	(0.18, 2.43)	0.70	(0.19, 2.62)	0.60	(0.15, 2.39)
I-ADL dependency: ^e^								
Low dependency			1.00		1.00		1.00	
High dependency			0.70	(0.21, 2.27)	0.61	(0.19, 2.00)	0.56	(0.17, 1.85)
Not assessed			1.73	(0.37, 8.23)	1.55	(0.32, 7.54)	1.64	(0.32, 8.25)
Need for help with social participation: ^e^								
Low dependency			1.00		1.00		1.00	
High dependency			1.65	(0.63, 4.34)	1.53	(0.57, 4.09)	1.70	(0.63, 4.59)
Not assessed			1.76	(0.85, 3.64)	1.67	(0.80, 3.50)	1.66	(0.78, 3.53)
Need for help with memory: ^e^								
Low dependency			1.00		1.00		1.00	
High dependency			5.36 ***	(2.26, 12.71)	4.74 ***	(1.96, 11.44)	5.38 ***	(2.17, 13.36)
Not assessed			1.12	(0.51, 2.46)	1.18	(0.53, 2.63)	1.14	(0.50, 2.59)
LOS (days) ^f^					1.01	(0.98, 1.05)	1.02	(0.98, 1.06)
Healthcare use index score ^g^					2.15 ***	(1.55, 2.98)	2.18 ***	(1.56, 3.04)
Education: ^h^								
Primary education							1.00	
Secondary education							1.32	(0.79, 2.22)
Tertiary education							1.56	(0.76, 3.22)
Income (in NOK): ^i^								
100,000-199,999							1.14	(0.65, 2.00)
200,000-299,999							1.00	
300,000-399,999							1.54	(0.86, 2.78)
400,000+							1.07	(0.50, 2.28)
Wealth (in NOK): ^i^								
≤ 0							0.94	(0.02, 49.79)
1-199,999							1.75	(0.84, 3.64)
200,000-499,999							1.41	(0.73, 2.73)
500,000-999,999							0.74	(0.41, 1.33)
1,000,000+							1.00	
Pseudo R^2^	0.080		0.180		0.212		0.227	
AIC	698.8		654.2		634.9		642.1	
BIC	765.0		782.3		771.8		818.8	
AUC	0.69		0.78		0.80		0.81	
Observations	612		612		612		612	

*Notes*: ** *p* < 0.01, *** *p* < 0.001; ^a^ all models include fixed-effects for Oslo’s boroughs (not shown); ^b^ OR = Odds Ratio; ^c^ CI = Confidence Interval; ^d^ Fracture of: shaft of femur, lower end of femur, multiple fractures of femur, other/unspecified parts of femur; ^e^ within 30 days after hospital discharge for HF; ^f^ of the index hospital episode; ^g^ measures change in the scope of municipal healthcare services received between the first five months post-discharge and the month prior to the index admission; ^h^ in the year of HF; ^i^ in the year prior to the HF

In Model 1, the odds of institutionalization was 74 % lower for patients who received rehabilitation outside of an institution compared to patients who received rehabilitation during a short-term stay in an institution (*p* < 0.001).

In Model 2, we also included controls for patients’ gender and age, health characteristics and functional level. Here, the effect associated with rehabilitation outside of an institution was reduced (to 69 % lower odds of institutionalization), but it was still significant. There was no significant gender difference in the odds of institutionalization, but a one-year increase in age was associated with a statistically significant 8 % increase in the odds of institutionalization (*p* < 0.001). There was no significant difference in the odds of institutionalization between patients with comorbidities versus those without, or between patients with a fracture of the neck of femur compared to patients with some other type of fracture. There was no significant effect of high dependency in P-ADLS, I-ADLs or in need for help with social participation, compared to low dependency–but patients who were dependent on help with memory had 5.36 (*p* < 0.001) times higher risk compared to patients who were independent in this area.

An additional set of variables that described patients’ use of healthcare services was added in Model 3. There was no significant effect of increased hospital LOS. However, the risk of institutionalization was elevated for patients who experienced a greater need for support after the injury. Specifically, a one unit increase in the healthcare use index score, which represents having used more municipal healthcare services during the post-fracture period compared to the pre-fracture period, increases the probability of living in a nursing home at 6–12 months after the HF by 115 % (*p* < 0.001).

The full model, Model 4, also included variables describing patients’ socioeconomic status—i.e., education, income, and wealth. For each of these variables, there were no statistically significant group differences in the risk of institutionalization. In Model 4, the odds of institutionalization was 58 % lower for patients who had received rehabilitation outside of an institution (*p* < 0.01) compared to those who had received a short-term rehabilitation stay in an institution.

Supplementary file 2 contains a sensitivity analysis including 172 of the observations that were originally excluded because we could not categorize them as either living at home or living in an institution at 6–12 months after the HF (see step 3 in Fig. 1).

## Discussion

This study investigated the effect of rehabilitation setting on HF patients’ risk of institutionalization. Specifically, we analyzed whether receiving rehabilitation outside of an institution—in contrast to a short-term rehabilitation stay in an institution—was associated with a lower risk of being admitted to a nursing home for LTC. Our findings demonstrated that the odds of institutionalization was 58 % lower for patients who had received rehabilitation outside of an institution. The strong effect remained, even after controlling for patients’ age, gender, health characteristics, healthcare use, and socioeconomic status. Furthermore, we saw that older age, high post-fracture ADL dependency in need for help with memory, and increased use of municipal healthcare services were significant predictors of institutionalization.

There is a growing trend towards deinstitutionalization in Norway, such that an increasing number of patients are receiving rehabilitation and other healthcare services at home and in their local community post-discharge from hospital [38, 39]. This trend is driven in part by financial pressures faced by the government to deliver healthcare services to a large number of older adults with chronic conditions [51, 52]. The question then arises as to whether treating patients at lower levels of care addresses the parallel challenges of reducing costs and safeguarding patient outcomes. This study sought to answer the latter, specifically with regard to risk of institutionalization in a nursing home—which is linked to a higher probability of death not only due to the selection of patients in poor health into nursing homes [53–55]. While our results imply that the setting in which HF patients receive rehabilitation does affect their likelihood of being institutionalized—and in particular, that rehabilitation outside of an institution is associated with an increased likelihood of remaining at home (community)—this study has some weaknesses which require us to generalize the findings with caution.

Our results should not be seen as evidence of a causal effect. Despite our best efforts at risk adjustment, we cannot rule out the possibility that some of the favorable effect we saw from receiving rehabilitation outside of an institution is due to unobserved heterogeneity in our sample. In a real-life setting, the decision on what type of rehabilitation a patient should receive is not random. Healthcare professionals’ evaluation of the patient—including the assessment of their rehabilitation potential, home environment, and social support—is crucial for the choice of discharge location and the overall patient pathway. The large difference in the odds of institutionalization is presumably influenced by differences between the two patient groups that affect both the type of rehabilitation they receive and their odds of being institutionalized later on. The lack of a measure to capture the criteria informing the decision about what type of rehabilitation a patient should receive is the primary limitation of the current study. Some of our key measures of patients’ health status were limited. Specifically, we know that our measure of comorbidities is an underestimation, because it is based on hospital admissions only—and not GP consultations or use of prescription drugs, which could have captured less severe health impairments. Additionally, there are some important weaknesses in our ADL variables. First, we did not control for time between the HF and the ADL assessment. Scores collected a full month after hospital discharge may represent a different need for help than scores obtained quickly after the HF. Furthermore, ADL functional status was not assessed for a large proportion of our sample. The reason for this appears to be that there is a lag in the municipality’s registrations/updates of ADL scores. Although it is unfortunate for the data quality, it makes sense—since providing the necessary services to the user should be prioritized over data registration. Nevertheless, it means that we must interpret the results with caution, as the unknown ADL status could falsely inflate the protective effect of rehabilitation outside of an institution on institutionalizations at 6–12 months. We also lacked data that may be important for isolating the effect of rehabilitation setting on institutionalization. For example, we did not control for fracture-related complications, type of HF surgery, or whether or not patients lived alone—which is an indicator for social support. These factors can both affect which patients are eligible for rehabilitation at home, as well of their risk of institutionalization. These are important data needs and considerations for future studies on this topic.

Understanding the mechanism through which our finding is produced is important. The city of Oslo, which is the setting for this study, is comprised of 15 boroughs that have different models of rehabilitation (e.g., in terms of the depth and breadth of the services delivered, and by whom). Given this variation, we are unable to precisely define the intervention “rehabilitation outside an institution” and explore questions regarding its components and how they might explain our findings. Our regression models did however include fixed-effects for Oslo’s boroughs and thus adjusted for the effects of unmeasured variation (e.g., in home-based rehabilitation) across the boroughs on the risk of institutionalization.

Despite some of the aforementioned challenges, our study adds to the existing but sparse literature that links home-based rehabilitation with positive patient outcomes. All else being equal, receiving rehabilitation outside of an institution rather than a short-term rehabilitation stay in an institution appears to protect against future risk of institutionalization. A possible explanation for this is that the home-setting likely encourages and facilitates the tailoring of rehabilitative care and training to patients’ everyday lives [5, 16, 19]. There is some inconclusive evidence that links home-based reablement care to improved functional status among older adults, compared to usual care [22, 24, 56]. All else being equal: it is reasonable to expect that rehabilitation provided to older adults at home, and aimed at promoting their functioning and independence in activities of daily life within their home and community settings, may be more advantageous in supporting older adults to continue living at home compared to short-term rehabilitation provided in an institution.

The data were collected to 2014, and consequently, the allocation practices for the different types of rehabilitation may have changed as ‘reablement’ and similar rehabilitation models have gained a firm foothold. Further research on more recent data can provide even clearer insights into how this type of rehabilitation performs in a real-life setting.

## Conclusions

This study demonstrates that the setting in which HF patients receive rehabilitation is associated with their likelihood of institutionalization. The strong effect remains, even after controlling for patients’ age, gender, health characteristics and level of function, healthcare use, and socioeconomic status. Patients who receive rehabilitation outside of an institution are 58 % less likely to live in a nursing home for LTC at 6–12 months after the HF, compared to patients who receive a short-term rehabilitation stay in an institution. Our findings, while tentative pending more rigorous study designs, suggest that providing rehabilitation at home may be favorable in terms of reducing risk of institutionalization for HF patients. These findings could potentially inform clinical work in a scenario where HF patients are assessed (functional and health status, family and living situation) pre- and post-treatment to identify those for whom rehabilitation at home would be most appropriate and beneficial. Recipients of home-based rehabilitation would then receive care in their preferred setting (at home) and in a manner that promotes their functional status, including with regard to ADLs in their home settings. Short- and long-term institutional places and resources in the municipal primary care would then be reserved for patients in need of such support. Given the high costs of institutional care, providing the right patients with the right care in the right setting can potentially save costs and safeguard patient outcomes.

## Supplementary Information

**Additional file 1.**

**Additional file 2.**

## Data Availability

The registry datasets used are subject to strict anonymity, confidentiality and data protection laws. Due to these regulations and the necessity to ensure that they are not compromised or breached, publication of the dataset is not possible. Access to the data used in the study can be obtained from Oslo Municipality; the Norwegian Patient Registry; Norway Control and Payment of Health Reimbursement (KUHR) database; the Norwegian Cause of Death Registry, and Statistics Norway’s database FD-Trygd.

## Abbreviations

HF: Hip fracture
LTC: Long-term care
OR: Odds ratio
CI: Confidence interval
ADLs: Activities of daily living
P-ADLs: Personal ADLs
I-ADLs: Instrumental ADLs
LOS: Length of stay
ICD-10: International Statistical Classification of Diseases and Related Health Problems, 10th revision
GP: General practitioner
PID: Personal identification number
SD: Standard deviation

## Footnotes

1 The variable covers 90–365 days back in time, depending on when in the year 2008 the patient was admitted to hospital for HF.

2 The two neighboring boroughs were merged due to few cases.
