# Supplementary material for: Does rehabilitation setting influence risk of institutionalization? A register-based study of hip fracture patients in Oslo, Norway
Source: BMC Health Serv Res. 2021 Jul 9;21:678. doi: 10.1186/s12913-021-06703-x (PMC8268388; doi:10.1186/s12913-021-06703-x)
Supplement: Supplementary file 1 — Additional file 1. [file 12913_2021_6703_MOESM1_ESM.docx]

Additional file #1 to “Does rehabilitation setting influence risk of institutionalization? A register-based study of hip fracture patients in Oslo, Norway”

| Table A1: Comparison of characteristics of the analytic sample (n=612) and the censored observations (n=1,624) | | | | | |
| --- | --- | --- | --- | --- | --- |
|  | **Analytic sample**  **(n=612)** | | **Censored observations**  **(n=1,624)** | | |
|  | Mean or percent | SD ^a^ | Mean or percent | SD ^a^ | *n* (percent missing data) |
| Place of residence at 6-12 months after index admission: |  |  |  |  | 1,624 (0) |
| Home | 71.9 |  | 55.8 |  |  |
| Nursing home | 28.1 |  | 9.4 |  |  |
| Mixed (both at home and in nursing home) | Excluded |  | 13.5 |  |  |
| Unknown | Excluded |  | 21.3 |  |  |
| Rehabilitation setting: ^b^ |  |  |  |  | 1,624 (0) |
| Outside institution | 49.0 |  | 10.0 |  |  |
| In institution | 51.0 |  | 14.0 |  |  |
| In both settings | Excluded |  | 3.3 |  |  |
| In none of the two settings | Excluded |  | 72.5 |  |  |
| Death within 365 days from index admission | Excluded |  | 19.8 |  | 1,624 (0) |
| Lived in a nursing home prior to the HF | Excluded |  | 15.4 |  | 1,624 (0) |
| Male (vs. female) | 21.1 |  | 28.2 |  | 1,624 (0) |
| Age (years) | 82.4 | (9.0) | 80.0 | (15.2) | 1,624 (0) |
| One or more comorbidities (vs. none) | 12.9 |  | 9.8 |  | 1,624 (0) |
| HF diagnosis: |  |  |  |  | 1,624 (0) |
| Fracture of neck of femur | 51.0 |  | 50.2 |  |  |
| Pertrochanteric fracture | 37.4 |  | 34.4 |  |  |
| Subtrochanteric fracture | 5.1 |  | 4.6 |  |  |
| Other ^c^ | 6.5 |  | 10.8 |  |  |
| P-ADL dependency: ^d^ |  |  |  |  | 1,624 (0) |
| Low dependency | 18.1 |  | 7.1 |  |  |
| High dependency | 42.0 |  | 25.4 |  |  |
| Not assessed | 39.9 |  | 67.5 |  |  |
| I-ADL dependency: ^d^ |  |  |  |  | 1,624 (0) |
| Low dependency | 6.9 |  | 3.4 |  |  |
| High dependency | 52.1 |  | 27.3 |  |  |
| Not assessed | 41.0 |  | 69.3 |  |  |
| Help with social participation: ^d^ |  |  |  |  | 1,624 (0) |
| Low dependency | 25.8 |  | 10.5 |  |  |
| High dependency | 6.5 |  | 5.1 |  |  |
| Not assessed | 67.6 |  | 84.4 |  |  |
| Help with memory: ^d^ |  |  |  |  | 1,624 (0) |
| Low dependency | 24.2 |  | 9.7 |  |  |
| High dependency | 10.3 |  | 6.9 |  |  |
| Not assessed | 65.5 |  | 83.4 |  |  |
| LOS (days) ^e^ | 11.4 | (5.3) | 10.2 | (8.5) | 1,624 (0) |
| Healthcare use index score ^f^ | 1.3 | (0.7) | 0.6 | (0.8) | 1,624 (0) |
| Education: ^g^ |  |  |  |  | 1,580 (2.7) |
| Primary education | 34.3 |  | 34.0 |  |  |
| Secondary education | 49.2 |  | 44.1 |  |  |
| Tertiary education | 16.5 |  | 22.0 |  |  |
| Income (in NOK): ^h^ |  |  |  |  | 1,624 (0) |
| ≤99,999 | Excluded |  | 3.0 |  |  |
| 100,000-199,999 | 27.0 |  | 27.0 |  |  |
| 200,000-299,999 | 38.4 |  | 33.9 |  |  |
| 300,000-399,999 | 21.2 |  | 18.5 |  |  |
| 400,000+ | 13.4 |  | 17.5 |  |  |
| Wealth (in NOK): ^h^ |  |  |  |  | 1,624 (0) |
| ≤0 | 0.3 |  | 3.1 |  |  |
| 1-199,999 | 16.0 |  | 20.6 |  |  |
| 200,000-499,999 | 18.1 |  | 14.7 |  |  |
| 500,000-999,999 | 29.1 |  | 22.0 |  |  |
| 1,000,000+ | 36.4 |  | 39.6 |  |  |
| Borough: |  |  |  |  | 1,595 (1.8) |
| Gamle Oslo | 3.4 |  | 5.3 |  |  |
| Grünerløkka | 7.4 |  | 5.6 |  |  |
| Sagene | 6.9 |  | 5.1 |  |  |
| St. Hanshaugen | 3.8 |  | 4.5 |  |  |
| Frogner | 8.8 |  | 10.3 |  |  |
| Ullern | 4.7 |  | 5.8 |  |  |
| Vestre Aker | 8.7 |  | 8.5 |  |  |
| Nordre Aker | 11.6 |  | 7.5 |  |  |
| Bjerke | 2.9 |  | 5.0 |  |  |
| Grorud + Stovner | 4.2 |  | 10.9 |  |  |
| Alna | 5.9 |  | 7.8 |  |  |
| Østensjø | 15.7 |  | 11.0 |  |  |
| Nordstrand | 12.7 |  | 10.0 |  |  |
| Søndre Nordstrand | 3.3 |  | 2.8 |  |  |
| ***Notes:*** ^a^ SD = standard deviation; ^b^ within 30 days post-discharge; ^c^ Fracture of: shaft of femur, lower end of femur, multiple fractures of femur, other/unspecified parts of femur;  ^d^ within 30 days after hospital discharge for HF; ^e^ of the index hospital episode; ^f^ measures change in the scope of municipal healthcare services received between the first five months post-discharge and the month prior to the index admission; ^g^ in the year of HF; ^h^ in the year prior to the HF. | | | | | |

Table A1 shows characteristics of the analytic sample and the censored observations that was excluded (see Figure 1). The table includes descriptive statistics for all study variables, as well as for the exclusion criteria that were used to define the analytic sample.

Due to our exclusion criteria, two continuous variables were estimated differently for the censored observations than for the analytic sample:

- Age: The estimate of mean age shown in Table A1 includes 60 observations aged <50 years old (range: 1-103; an exclusion criterion for the analytic sample). Among those aged 50 years and older, the mean age was 82.1 years.
- LOS: The estimate of average LOS shown in Table A1 includes 64 patients with LOS of >30 days (range: 2-75; an exclusion criterion for the analytic sample). Among those with a LOS of ≤30 days, the average LOS was 9.0 days.
