# Supplementary material for: Does rehabilitation setting influence risk of institutionalization? A register-based study of hip fracture patients in Oslo, Norway
Source: BMC Health Serv Res. 2021 Jul 9;21:678. doi: 10.1186/s12913-021-06703-x (PMC8268388; doi:10.1186/s12913-021-06703-x)
Supplement: Supplementary file 2 — Additional file 2. [file 12913_2021_6703_MOESM2_ESM.docx]

Additional file #2 to “Does rehabilitation setting influence risk of institutionalization? A register-based study of hip fracture patients in Oslo, Norway”

**Sensitivity analysis**

| *Table A2: Sensitivity analysis*  Logistic regression of institutionalization one year after index admission for hip fracture on post-fracture rehabilitation setting ^a^ | | | | | | | | |
| --- | --- | --- | --- | --- | --- | --- | --- | --- |
|  | **Model 1** | | **Model 2** | | **Model 3** | | **Model 4** | |
|  | **OR ^b^** | **95% CI ^c^** | **OR ^b^** | **95% CI ^c^** | **OR ^b^** | **95% CI ^c^** | **OR ^b^** | **95% CI ^c^** |
| Rehabilitation outside institution (vs. in institution) | 0.41 *** | (0.27, 0.61) | 0.46 *** | (0.30, 0.72) | 0.64 | (0.40, 1.01) | 0.64 | (0.40, 1.02) |
| Male (vs. female) |  |  | 0.96 | (0.63, 1.47) | 0.94 | (0.61, 1.45) | 0.93 | (0.60, 1.46) |
| Age (years) |  |  | 1.07 *** | (1.04, 1.09) | 1.06 *** | (1.03, 1.09) | 1.06 *** | (1.04, 1.09) |
| One or more comorbidities (vs. none) |  |  | 1.84 * | (1.16, 2.93) | 1.82 * | (1.14, 2.91) | 1.83 * | (1.14, 2.94) |
| HF diagnosis: |  |  |  |  |  |  |  |  |
| Fracture of neck of femur |  |  | 1.00 |  | 1.00 |  | 1.00 |  |
| Pertrochanteric fracture |  |  | 1.01 | (0.71, 1.45) | 0.95 | (0.66, 1.36) | 0.95 | (0.65, 1.37) |
| Subtrochanteric fracture |  |  | 1.04 | (0.49, 2.23) | 0.97 | (0.44, 2.14) | 0.96 | (0.43, 2.14) |
| Other ^d^ |  |  | 0.92 | (0.44, 1.93) | 0.89 | (0.41, 1.90) | 0.88 | (0.41, 1.91) |
| P-ADL dependency: ^e^ |  |  |  |  |  |  |  |  |
| Low dependency: |  |  | 1.00 |  | 1.00 |  | 1.00 |  |
| High dependency |  |  | 1.09 | (0.59, 2.01) | 1.03 | (0.55, 1.91) | 1.01 | (0.54, 1.89) |
| Not assessed |  |  | 1.06 | (0.41, 2.77) | 1.04 | (0.39, 2.74) | 0.96 | (0.36, 2.58) |
| I-ADL dependency: ^e^ |  |  |  |  |  |  |  |  |
| Low dependency |  |  | 1.00 |  | 1.00 |  | 1.00 |  |
| High dependency |  |  | 0.59 | (0.25, 1.40) | 0.57 | (0.24, 1.36) | 0.56 | (0.23, 1.34) |
| Not assessed |  |  | 0.79 | (0.25, 2.49) | 0.79 | (0.25, 2.51) | 0.82 | (0.25, 2.63) |
| Need for help with social participation: ^e^ |  |  |  |  |  |  |  |  |
| Low dependency |  |  | 1.00 |  | 1.00 |  | 1.00 |  |
| High dependency |  |  | 1.45 | (0.66, 3.21) | 1.39 | (0.62, 3.09) | 1.35 | (0.60, 3.02) |
| Not assessed |  |  | 1.43 | (0.79, 2.58) | 1.40 | (0.76, 2.56) | 1.38 | (0.75, 2.53) |
| Need for help with memory: ^e^ |  |  |  |  |  |  |  |  |
| Low dependency |  |  | 1.00 |  | 1.00 |  | 1.00 |  |
| High dependency |  |  | 4.34 *** | (2.15, 8.79) | 4.03 *** | (1.97, 8.26) | 4.32 *** | (2.09, 8.93) |
| Not assessed |  |  | 1.41 | (0.76, 2.62) | 1.44 | (0.76, 2.71) | 1.44 | (0.76, 2.73) |
| LOS (days) ^f^ |  |  |  |  | 1.01 | (0.98, 1.05) | 1.01 | (0.98, 1.05) |
| Healthcare use index score ^g^ |  |  |  |  | 1.98 *** | (1.52, 2.58) | 2.00 *** | (1.53, 2.62) |
| Education: ^h^ |  |  |  |  |  |  |  |  |
| Primary education |  |  |  |  |  |  | 1.00 |  |
| Secondary education |  |  |  |  |  |  | 1.09 | (0.72, 1.65) |
| Tertiary education |  |  |  |  |  |  | 1.06 | (0.60, 1.89) |
| Income (in NOK): ^i^ |  |  |  |  |  |  |  |  |
| 100,000-199,999 |  |  |  |  |  |  | 1.03 | (0.65, 1.63) |
| 200,000-299,999 |  |  |  |  |  |  | 1.00 |  |
| 300,000-399,999 |  |  |  |  |  |  | 1.23 | (0.77, 1.97) |
| 400,000+ |  |  |  |  |  |  | 0.95 | (0.51, 1.79) |
| Wealth (in NOK): ^i^ |  |  |  |  |  |  |  |  |
| ≤0 |  |  |  |  |  |  | 1.26 | (0.16, 10.09) |
| 1-199,999 |  |  |  |  |  |  | 1.13 | (0.62, 2.07) |
| 200,000-499,999 |  |  |  |  |  |  | 1.35 | (0.80, 2.28) |
| 500,000-999,999 |  |  |  |  |  |  | 0.78 | (0.48, 1.24) |
| 1,000,000+ |  |  |  |  |  |  | 1.00 |  |
| Pseudo R^2^ | 0.047 |  | 0.122 |  | 0.151 |  | 0.157 |  |
| AIC | 952.0 |  | 907.0 |  | 883.3 |  | 895.7 |  |
| BIC | 1021.9 |  | 1042.3 |  | 1027.9 |  | 1082.3 |  |
| AUC | 0.65 |  | 0.74 |  | 0.76 |  | 0.76 |  |
| Observations | 784 |  | 784 |  | 784 |  | 784 |  |
| ***Notes:*** * *p*<0.05, ** *p*<0.01, *** *p*<0.001; ^a^ all models include fixed-effects for Oslo’s boroughs (not shown); ^b^ OR = Odds Ratio; ^c^ CI = Confidence Interval; ^d^ Fracture of: shaft of femur, lower end of femur, multiple fractures of femur, other/unspecified parts of femur;  ^e^ within 30 days after the HF; ^f^ of the index hospital episode; ^g^ measures change in the scope of municipal healthcare services received between the first five months post-discharge and the month prior to the index admission; ^h^ in the year of HF; ^i^ in the year prior to the HF. | | | | | | | | |

When we defined the sample for our main analysis, we excluded 369 patients because we could not categorize them as either living at home or living in an institution at 6-12 months after the HF, following our definition of the variable ‘institutionalization’.

In our main analysis, the independent variable ‘institutionalization’ was coded as follows: 0=the patient was living at home for the whole period; 1=the patient was living in a nursing home or was in an institution for treatment/rehabilitation for the entire period.

Of the 369 patients, the majority would have been excluded regardless—based on the other criteria we used to define the sample (most of them due to death), but for some of them (n=172) it was possible to ascertain where they were one year after HF (more specifically, on day 365).

We conducted a sensitivity analysis to investigate whether or not the inclusion of these patients in the sample would affect the result.

The sample in the sensitivity analysis is comprised of 784 observations. This includes the original analytic sample (612 observations), and 172 new observations.

The dependent variable in the sensitivity analysis is therefore a different operationalization of the variable ‘institutionalization’. Here, 1=the patient was in a nursing home an institution for treatment/rehabilitation on day 365 after the HF (defined as “living in an institution”), 0=the patient was at home on day 365 after the HF (defined as “living at home”).

As shown in Table A2, the effect of rehabilitation setting is no longer significant in Models 3 and 4, compared to in our main results (Table 3).

In the sensitivity analysis, patients with one or more comorbidities have an elevated risk of being institutionalized one year after the HF, compared to patients without any registered comorbidities. This variable was not significant in any of the models in our main results.

Like in our main results, there are still significant effects of higher age, being dependent on help with memory, and of receiving more municipal healthcare services after the HF in the sensitivity analysis.

The result of this sensitivity analysis could be expected. While our main analyses captured patients who move to a nursing home relatively quickly after the HF, our sensitivity analysis captures patients who live longer at home before ending up in a nursing home. In Norway, very few patients move back home after coming to a nursing home.

Furthermore, as time passes, the effect of the HF and the setting in which the patient was rehabilitated will become weaker. In our main analysis, we looked at the effect of rehabilitation setting on institutionalization at 6-12 months after HF. It is not surprising that we do not see a similar effect of rehabilitation setting on institutionalization on day 365 after HF.
